# Impact of Motivational Interviewing on Self-Management in Patients With Type 2 Diabetes: Protocol for a Pilot Randomized Controlled Trial

**DOI:** 10.2196/15709

**Published:** 2020-03-31

**Authors:** Man Kin Wong, Sai Yip Ronald Cheng, Tsun Kit Chu, Fung Yee Lam, Shiu Kee Lai, Kai Chung Wong, Jun Liang

**Affiliations:** 1 Department of Family Medicine and Primary Health Care Tuen Mun Hospital New Territories West Cluster New Territories Hong Kong

**Keywords:** motivational interviewing, diabetes, nurse, usual care, self-management, self-care

## Abstract

**Background:**

The nonpharmacological approach to diabetic control in patients with diabetes focuses on a healthy diet, physical activity, and self-management. Therefore, to help patients change their habits, it is essential to identify the most effective approach. Many efforts have been devoted to explain changes in or adherence to specific health behaviors. Such efforts have resulted in the development of theories that have been applied in prevention campaigns and include brief advice and counseling services. Within this context, motivational interviewing (MI) has proven to be effective in changing health behaviors for specific cases. However, stronger evidence is needed on the effectiveness of MI in treating chronic pathologies such as diabetes.

**Objective:**

This study will obtain preliminary data on the impact of a nurse-led MI intervention in improving glycemic control, as well as clinical, psychosocial, and self-care outcomes for individuals with type 2 diabetes mellitus when compared with usual care, with the aim of improving diabetic control in patients with diabetes.

**Methods:**

An open, two-arm, parallel, randomized controlled, pilot exploratory trial will be performed. Two government outpatient clinics in the New Territories West Cluster in Hong Kong will be involved. In total, 20 to 25 participants will be invited in each arm. Intervention participants will receive face-to-face MI interventions in addition to their usual care from the clinic. Control participants will only receive usual care. Outcomes are assessed at baseline, 6 months, and 12 months. The primary outcome measure is glycated hemoglobin levels. Secondary outcomes include blood pressure, BMI, hip and waist circumference, fasting blood, and psychosocial and self-care measures.

**Results:**

This study is currently underway with funding support from the Hong Kong College of Family Physician Research Seed Fund 2017.

**Conclusions:**

MI skills constitute the main strategies primary care nurses use on their patients. Having economical, simple, effective, and applicable techniques is essential for primary care professionals to help their patients change their lifestyle and improve their health. This study will provide scientific evidence on the effectiveness of MI. It will be performed with strict control over the data collection, ensuring the maintenance of therapeutic integrity.

**Trial Registration:**

Centre for Clinical Research and Biostatistics CUHK_CCRB00614; https://tinyurl.com/v9awzk6

**International Registered Report Identifier (IRRID):**

DERR1-10.2196/15709

## Introduction

### Background

Self-management of diabetes mellitus (DM) requires that patients can reconcile their resources and preferences with the standard therapeutic regimen for diabetes, a task that can be a challenging for many patients [1,2]. It has been widely accepted that diabetes education is not only required in the first few months following diagnosis, but is an important component of ongoing diabetes care because of the demanding self-care requirements, which requires multiple daily decisions to balance diet, physical activity, and medications [3]. There has been a keen interest in examining the impact that different kinds of patient education programs have on diabetes self-management. The findings of several meta-analyses from randomized controlled trials (RCTs) provide extensive evidence for the effectiveness of behavioral and educational intervention on fasting blood glucose and glycated hemoglobin (HbA_1c_). However, the long-term effects of such interventions are uncertain [4-11]. Knowledge about the effectiveness of behavioral and educational interventions on other diabetes-related outcome measures including blood pressure, lipid profile, body weight, self-management skills, health behavior, and psychosocial aspects are currently inconclusive [3,10-13]. There are few studies that have examined the impact of these psychological interventions among Chinese patients with diabetes. Critical assessment regarding the impact of behavioral and educational programs requires further research that is based on rigorous methods from high quality studies and are adequately powered as well as furnished with long-term follow-ups. The precise magnitude of the effectiveness should be examined, well-defined, and specific to the service recipients when designed [12-14].

Motivational interviewing (MI), described by Rollnick and Miller [15], is a well-defined and scientifically tested method of client counselling that has successfully been used to elicit and sustain a person’s behavior changes in a number of health care areas. A recent review of MI showed improvements in health behaviors such as diet and exercise in patients with diabetes [16]. However, studies on the effectiveness of MI have not been able to offer unanimous conclusions on clinical [17-23] and psychosocial attributes [18,21,24-27] in patients with diabetes. Previous reviews emphasize the need for studies of high methodological quality and adequate power to explore the effect of MI on glycemic control and well-being in patients with diabetes [14,16,28].

Given the fast pace of consultation flow in Hong Kong, where consultation time for a general practitioner is around 5 minutes for each patient in government outpatient clinics (GOPC), it is not possible for doctors to carry out MI. Management of diabetes involves a multidisciplinary approach in which allied health professionals such as dietitians, occupational therapists, and physiotherapists are also important team members. Our study will be novel as it is the first RCT using samples of Chinese patients with diabetes. In addition, we invited trained nurses to participate in our intervention.

### Knowledge Gap

Evidence-based MI research in various health care aspects is available abroad, but there is a dearth of such research in Hong Kong. It is important to build up local data on MI research.

### Objectives

This project aims to obtain preliminary data regarding the impact of MI on diabetes control compared with normal care among patients seen in primary care settings. The outcomes of interest include glycemic control (as measured by HbA_1c_), blood pressure, BMI, hip and waist circumference, fasting blood, and psychosocial and self-care measures.

The findings could inform the efficacy of MI for diabetes management in patients in family practices and contribute to better disease control.

## Methods

### Study Design

A pilot multicenter, parallel-group, RCT will be performed. It is an assessor-blinded study, involving two GOPCs in Hong Kong. They will be randomized with one allocated to an intervention group and the other to a control group.

Patients who attend the nurse-led clinic (NLC) in the intervention group will receive up to five individual counselling sessions based on MI in 1 year, in addition to their usual care.

A RCT design was chosen to minimize contamination between the control and intervention participants that could occur if participants in the same health service were randomized to different treatment groups. Furthermore, because the intervention will be targeted at changing the behavior of health professionals, once trained, treatment leakage could occur, as it will be difﬁcult for them to avoid using the intervention techniques with control participants.

### Subjects

#### Inclusion Criteria

Patients are eligible if they were diagnosed as having type 2 DM for at least 1 year and are 18 to 65 years of age with poor DM control (defined as HbA_1c_≥8). Patients who have or have not received maximum dosages of oral hypoglycemic agents are equally eligible.

#### Exclusion Criteria

Patients who are pregnant as well as those with severe debilitating diseases that preclude adherence to recommendations (eg, end stage cancer), cognitive deficits, or medical conditions rendering the individuals incapable of completing informed consent or participating in the study are excluded.

### Hypotheses

Our hypotheses are that MI can reduce HbA_1c_ levels, improve clinical and psychosocial outcomes, and increase diabetes self-care when compared with patients’ usual care.

### Recruitment Procedure

The patients will be informed while participating in the NLC. Advanced practice nurses (APNs) will assist participants in achieving the primary goal of treatment for HbA_1c_ levels less than 7.0%, which is the target defined by the Manual for Risk Assessment and Management Programme (Diabetes Mellitus) in the Primary Care Setting under the Hospital Authority of Hong Kong. This primary goal is applicable for both groups in the study.

After randomization, all outcome measures will be assessed at baseline, 6 months, and 12 months in both groups. Randomization is generated by a research randomizer with a 1:1 allocation ratio in both groups. Each GOPC is considered as a single unit of randomization.

### Sample Size Calculation

Sample size estimates were calculated using the ﬁxed number of clusters (2) available to the current study. The intracluster correlation coefﬁcient (ICC) accounts for the greater similarity of responses in patients within clusters compared with between clusters, and we could apply an ICC of 0.05 as typical in primary care settings [29]. The effect size for the primary outcome, HbA_1c_, was anticipated to be 0.32%, based on findings from a published meta-analysis [29]. Using these parameters, a sample size calculator for cluster-randomized trials [29] could estimate a total number of samples that would be required to achieve a power of 80%, while maintaining an alpha of 5% and a participant attrition rate of 20%.

However, since this is a pilot study, we will deploy a convenient sampling of 20 to 25 samples in each arm depending on resources. We anticipate performing a pragmatic cluster RCT in the future when resources are available so that sample size can be calculated with adequate power.

### Stratification and Randomization

**Figure 1:**
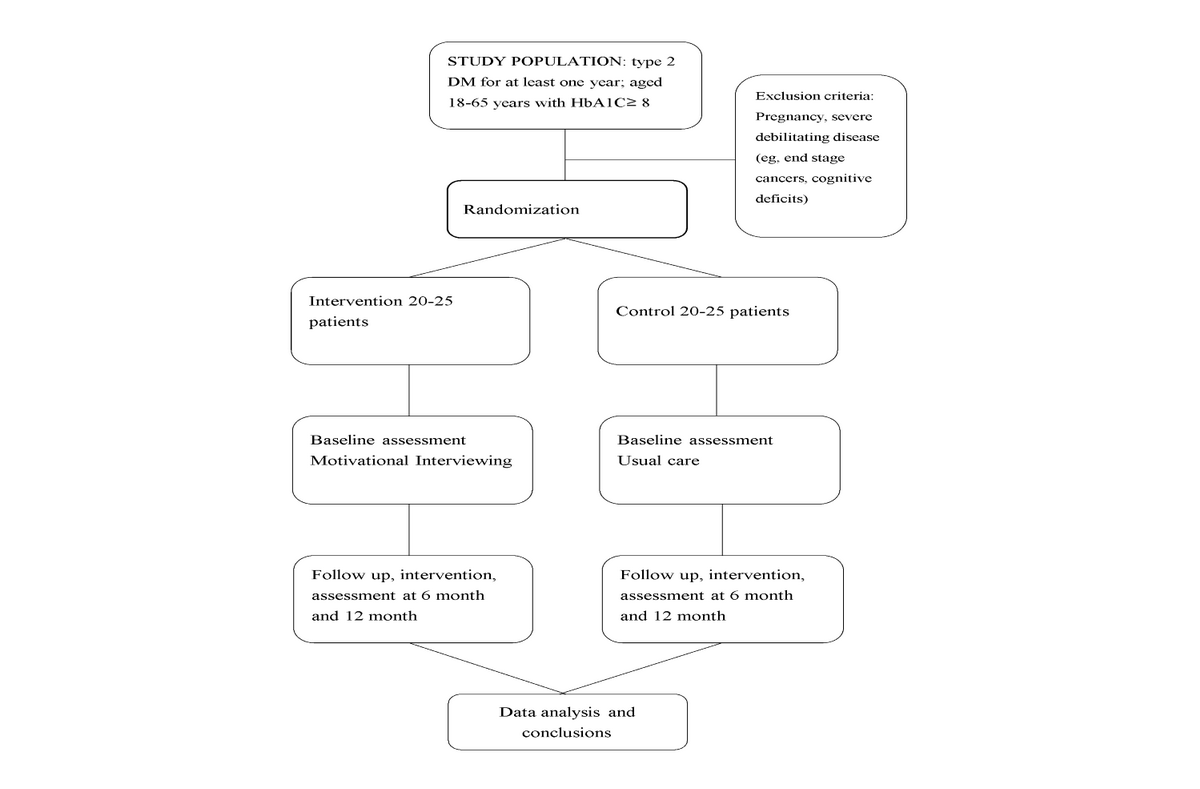
Trial participant flow. DM: diabetes mellitus; HbA_1c_: glycated hemoglobin.

Participants will be sampled by computerized random allocation software to achieve balance. The sampling procedure produces an ordered list of eligible participants. Then, recruiting ofﬁcers systematically invite eligible participants into the study. Once a participant declines participation, the recruiting ofﬁcer will move to the next person on the list. APNs will provide all participants an explanatory statement and consent form in person at their GOPC visit. Participant flow is shown in Figure 1.

### Usual Care

Medical treatment is not a part of the intervention. All participants, irrespective of participation in the intervention group or the control group, will undergo the same routine check-up at the GOPC in charge of their diabetes care. Biochemical tests and examinations will be performed during these visits in accordance with protocols.

Individual counselling and recommendations will be given based on the results of the examinations, biochemical tests, and self-monitoring of blood glucose. Renewal of prescribed medication will also be done at these check-up visits. Patients could be referred to individual counselling for diet modification, optimization of physical activity, smoking cessation if applicable, and minimization of alcohol use if required by their usual health care provider.

### Research Intervention

The theoretical approach of the intervention is based on the self-efficacy theory and MI spirit. Perceived self-efficacy is defined as people’s beliefs about their capabilities of producing designated levels of performance on exercising influence over events that affect their lives [30]. MI is used as a method to facilitate this process and is a directive counselling style for eliciting behavior change by helping patients to explore and resolve ambivalence [31,32]. In addition to their usual care, patients in the intervention group will receive a 1-year MI program consisting of up to five individual counselling sessions lasting approximately 30 to 45 minutes. Each participant in the intervention group is assigned an APN who has received training in MI. The style of the interview is standardized with the following components: seeking to understand the person’s frame of reference; expressing acceptance and affirmation; eliciting and selectively reinforcing the client’s own self-motivational statements of problem recognition, concern, desire and intention to change, and ability to change; exploring the client’s degree of readiness to change; and affirming the client’s freedom of choice and self-direction. Exploring readiness to change is used as a component of the therapeutic process and not an outcome. Each session follows a semistructured MI format, specifically developed for this intervention program. Participants may bring up any concerning issues related to diabetes self-care during the intervention sessions. The participants in the intervention group may be referred by the health care professional to individual counselling for lifestyle modification, which may include dietary changes, promotion of physical activity, and counseling on quitting smoking and minimizing alcohol consumption.

### Education of the Health Care Professionals Prior to the Intervention

A number of APNs will be educated to carry out MI, depending on funding and resources. They will be coached by trainers from the Motivational Interviewing Network of Trainers.

The theoretical and practical part of the education includes training in the key elements of MI, which is generally facilitated through eliciting change talk and exploring ambivalence about behavioral change while trying to examine discrepancies between the individual’s current behavior and core values or personal goals. The health care professionals will be introduced to MI methods including reflective listening and acknowledgement, so they will be able to clarify the patient’s goals and concerns, as well as elicit reasons for change using the patient’s own words. The role of the health care professionals is to coach and support the patients in discovering and developing their own resources for change and management at the patient’s request.

After the course, the health care professionals will be individually supervised by the MI trainer in 10 real patient situations. The supervision will include audiotaping and evaluation inspired by the Motivational Interviewing Treatment Integrity (MITI) coding system version 4.2.1 [33]. The MITI coding system is divided into a global rating and behavioral counts. The global rating is a 5-point Likert scale, where 1 indicates low competence in MI and 5 indicates high competence in MI. The behavior counts reveal MI behavior in proportion to all behavior, where a high percentage indicates a high competence in MI.

### Measurements and Outcomes

The primary outcome measure is HbA_1c_. Secondary clinical outcomes include systolic and diastolic blood pressure, weight, BMI, waist and hip circumference, and fasting blood samples (fasting plasma glucose, total cholesterol, triglyceride, high-density lipoprotein, and low-density lipoprotein). Additionally, secondary psychosocial and self-care behavior outcomes include: psychological distress (Kessler 10 [K10]; score range 10-50) [34], quality of life (QOL) (WHOQOL-BREF; domain score range 0-100) [35], diabetes self-care activities (Summary of Diabetes Self-Care Activities [SDSCA]; score range 0-7, representing number of days per week) [36], and diabetes management self-efﬁcacy (Chinese Diabetes Management Self-Efficacy Scale [C-DMSES]; score range 0-200) [37]. These surveys have been shown to demonstrate satisfactory psychometric properties.

K10 is a 10-item questionnaire intended to yield a global measure of distress based on questions about anxiety and depressive symptoms that a person has experienced in the most recent 4-week period.

The WHOQOL-BREF instrument comprises 26 items that measure the following broad domains: physical health, psychological health, social relationships, and environment. The WHOQOL-BREF is a shorter version of the original instrument that may be more convenient for use in large research studies or clinical trials. Both the self-administered and the interview version of the Hong Kong Chinese WHOQOL-BREF will be available [38].

The SDSCA measures frequency of self-care activity in the last 7 days for five aspects of the diabetes regimen: general diet (adherence to healthy diet), specific diet (ie, ate fruits and low-fat foods), foot care, blood-glucose testing, exercise, and taking recommended diabetes medication. Participants rate themselves from 0 to 7 on each item. The mean scores of the 11 items are then used to assess a participants’ self-care behavior.

C-DMSES assesses the extent that participants are confident they can self-monitor nutrition, blood sugar, foot exams, physical exercise, weight, and medical treatment. Participants rate themselves on an 11-point scale ranging from “0=can’t do at all” to “10=certain can do”. The mean scores of the 20 items are used to assess participants’ self-efficacy.

All patient level outcomes will be assessed at baseline, and again at 6 months and 12 months, during a clinical health check and an interviewer-administered questionnaire. All participants will be instructed to fast overnight for a minimum of 12 hours, and participant fasting times will be recorded prior to each blood test. When fasting times are insufﬁcient, participants will be asked to reschedule their appointment. Intervention and control groups will both undergo the same assessments, and all participants will be informed of their clinical results. Participants who are absent for outcome assessments will be contacted whenever possible by a phone call and asked to reschedule. Owing to the pragmatic nature of this trial, data collectors will not be blinded to group allocation; however, laboratory technicians will be blinded. Blood samples will be analyzed centrally at the Chemical Pathology Laboratories at Tuen Mun Hospital in Hong Kong.

### Fidelity

To externally verify that MI and usual care differ as expected, 2 out of 20 participants (10%) in both the MI and usual care sessions will be randomly selected for coding on adherence to MI principles using the MITI coding system by an expert independent coding group. Coders are blind to the study arm of the session and the study hypotheses. We anticipate that MI sessions will receive scores of 4 or higher on the 1 to 5 global rating and be significantly higher than usual care sessions on ratings of evocation, collaboration, empathy, and autonomous support. With respect to frequency measures for counselor behavior we expect MI sessions to have a higher reflection-to-question ratio and significantly fewer instances of giving information.

### Statistical Analysis

Descriptive statistics will be used to summarize the characteristics of the GOPCs and participants with regard to baseline characteristics and patterns of mean change over time. The primary analysis will examine the changes in HbA_1c_ at the 6- and 12-month follow-ups in comparison to the baseline. Secondary analyses will include all clinical, psychosocial, and self-care measures that are continuous outcomes. For data analyses, SPSS (IBM Corp, Armonk, NY) will be used. As this is a pilot study, nonparametric statistical tests will be used. The baseline values will be reported as means (SD). A *P*-value<.05 will be regarded as statistically significant.

### Ethical Considerations

This study is in compliance with the Helsinki Declaration and local legislation. Ethical approval was sought and granted by the New Territories West Cluster Clinical & Research Ethics Committee on July 3, 2018, with Ref. No.: NTWC/CREC/18038 at Tuen Mun Hospital in Hong Kong. All participants signed an informed consent from the nurses before participating in the study. This study is noncommercial.

To protect the interest of vulnerable subjects, confidentiality will be ensured according to the guidelines from the Hospital Authority in Hong Kong. For example, the data, including personal information, will be stored in encrypted files during and after the study. The investigators will be responsible for the safekeeping of the personal data, and only they will have access to the personal data during and after the study. The data will be kept in a locked cabinet for 5 years after the study. After completion of this storage period, the data files will be destroyed.

## Results

This study is currently underway with funding support from the Hong Kong College of Family Physician Research Seed Fund 2017. Participants could start the study at different times. All testing was completed in mid-November 2019. The results of the study will then be communicated via publications.

## Discussion

### Impact

Demonstrating which of the two methods tested here is more effective can have a major impact on clinical practices when designing and proposing clinical protocols for the management of diabetes. Clinical interviewing skills and health education are the essential strategies used by nurses with their patients. Obtaining scientific evidence on the effectiveness of MI in primary care through strict monitoring of the training methods and controlling the integrity of the therapies applied will allow us to have economical, brief, effective, and applicable techniques to help patients change their health habits and achieve better health outcomes.

### Limitations

This study design has several limitations. First, as the resources for this study are limited, we will not be able to collect a large sample size. Consequently, it might be more difficult to identify statistically significant intervention effects. This issue highlights the importance of preventing dropouts from the intervention. Dropouts will be prevented by sending telephone reminders to participants for follow-ups. In addition, to test our hypotheses, participants will need to fill out many questionnaires, which may cause higher levels of attrition.

### Conclusions

This study is one of the first attempts to assess the extent that the MI approach helps in overall diabetic care. The results obtained will help us understand the practical effectiveness of this approach, including its limitations and actual impact on Chinese patients with diabetes. If this method proves to be effective, our intention is to disseminate and promote that intervention used in APN sessions to promote better self-management.

## Appendix

Multimedia Appendix 1 CONSORT-eHEALTH checklist (V 1.6.1).

## Abbreviations

APN: advanced practice nurse
C-DMSES: Chinese Diabetes Management Self-Efficacy Scale
DM: diabetes mellitus
GOPC: government outpatient clinic
HbA_1c_: glycated hemoglobin
ICC: intracluster correlation coefficient
K10: Kessler 10
MI: motivational interviewing
MITI: Motivational Interviewing Treatment Integrity
NLC: nurse-led clinic
QOL: quality of life
RCT: randomized controlled trial
SDSCA: Summary of Diabetes Self-Care Activities.
